# Gender differences in experiential and facial reactivity to approval and disapproval during emotional social interactions

**DOI:** 10.3389/fpsyg.2015.01372

**Published:** 2015-09-22

**Authors:** Nicole Wiggert, Frank H. Wilhelm, Birgit Derntl, Jens Blechert

**Affiliations:** ^1^Division of Clinical Psychology, Psychotherapy, and Health Psychology, Department of Psychology, University of SalzburgSalzburg, Austria; ^2^Department of Psychiatry, Psychotherapy, and Psychosomatics, Medical Faculty, RWTH Aachen UniversityAachen, Germany; ^3^Translational Brain Medicine, Jülich Aachen Research AllianceAachen, Germany; ^4^Institute of Neuroscience and Medicine (INM-1), Research Center JülichJülich, Germany; ^5^Department of Psychiatry and Psychotherapy, University of TübingenTübingen, Germany; ^6^Centre for Cognitive Neuroscience, Department of Psychology, University of SalzburgSalzburg, Austria

**Keywords:** sex differences, social evaluation, emotion, facial electromyography, social interaction

## Abstract

Negative social evaluations represent social threats and elicit negative emotions such as anger or fear. Positive social evaluations, by contrast, may increase self-esteem and generate positive emotions such as happiness and pride. Gender differences are likely to shape both the perception and expression of positive and negative social evaluations. Yet, current knowledge is limited by a reliance on studies that used static images of individual expressers with limited external validity. Furthermore, only few studies considered gender differences on both the expresser and perceiver side. The present study approached these limitations by utilizing a naturalistic stimulus set displaying nine males and nine females (expressers) delivering social evaluative sentences to 32 female and 26 male participants (perceivers). Perceivers watched 30 positive, 30 negative, and 30 neutral messages while facial electromyography (EMG) was continuously recorded and subjective ratings were obtained. Results indicated that men expressing positive evaluations elicited stronger EMG responses in both perceiver genders. Arousal was rated higher when positive evaluations were expressed by the opposite gender. Thus, gender differences need to be more explicitly considered in research of social cognition and affective science using naturalistic social stimuli.

## Introduction

Gender differences have been fascinating scientists and lay people alike. Differences in physical characteristics such as height, weight, and brain size reveal a large body of literature (e.g., Faith et al., 2001; Rosenfeld, 2004; Cahill, 2006; Kirchengast and Marosi, 2008). Furthermore, the influence of cognitive abilities, behavior, and personality traits on gender differences is also well documented (e.g., Bussey and Bandura, 1999; Taylor et al., 2000; Chapman et al., 2007). However, gender differences in general reactivity to emotional stimuli such as affective pictures or films are less studied (e.g., Bradley et al., 2001; Derntl et al., 2009, 2010; Bagley et al., 2011). Research focusing on gender differences in *interpersonal emotional contexts* that examines both the stimulus side (expresser gender) and the perceiver side (participant gender) is even more scarce. It may reasonably be argued that this scarcity of research contrasts with the multitude of gender stereotypes regarding emotions in social interactions in the general population. The present study was designed to shed more light on this issue.

Social interactions encompass a coherent set of facial expressions, vocal components, and postural/gestural markers (Keltner and Haidt, 1999; Schweinberger and Schneider, 2014). Such rich communicative cues are thought to facilitate and disambiguate communication on multiple levels. Positively valenced social interactions expressing compliments, approval, and support are thought to signal sympathy and indicate affiliation or even attraction. In contrast, negatively valenced social interactions expressing criticism, disapproval, and discouragement repel the interaction and express antipathy or even hostility. Valenced social communication has a wide ranging psychological effect on the perceiver. For instance, positive evaluations may evoke emotions of happiness and pride and positively affect self-esteem (Fleming and Courtney, 1984). Negative social evaluations represent frequent and powerful stressors, eliciting anger, sadness, fear or embarrassment that may decrease self-esteem (Leary et al., 2006). In the following, we review existing behavioral, observational, and psychometric research on how gender modulates these response patterns while distinguishing between the expression (*expresser*) and the perception (*perceiver*) side.

Gender differences in the expression of emotions during social interactions (*expresser* side) have revealed a female susceptibility of emotional expressions (Dimberg and Lundquist, 1990; Kring and Gordon, 1998). Behaviorally, women have been shown to express positive evaluations like compliments more frequently than men (Holmes, 1993), possibly to enhance bonding with their interaction partners (Brown and Levinson, 1987) whereas men utilize compliments less often (Holmes, 1993). In contrast, negative social evaluations are used by both genders with similar frequency (Björkqvist et al., 1992). Furthermore, differences of emotion expressivity also depend on context and nature of the expressed emotion: women report more sadness, fear, shame or guilt and tend to engage more in related expressive behaviors in social encounters whereas men tend to exhibit more aggressive behavior than women when they feel angry (Biaggio, 1989; Pasick et al., 1990; Sharkin, 1993; Fischer et al., 2004a; Carré et al., 2013). Regarding the etiology of such gender differences both biological and cultural accounts have been put forward (Buck et al., 1974; Ekman and Friesen, 1982). Regarding the latter, the influence of social *display rules* may modulate an emotional response displayed by facial expressions (Buck, 1984). For instance, the expression of negative emotions might be more culturally acceptable for men than for women. Regarding the first (biological account), it is important to consider the evolutionary importance of mating situations and the role of positive expressions between opposite sex interaction partners to support affiliation and mating. Consistent with this account, emotional facial expressions of the opposite gender have been shown to result in faster detection times than emotional facial expressions of one’s own gender (Hofmann et al., 2006).

When perceiving negative emotions and evaluations (*perceiver* side) involving verbal aggression, women tend to attribute these to stress and the loss of self-control whereas men tend to view aggressive behavior as a tool to control others and demonstrate status (Campbell and Muncer, 1987). In response to positive evaluation, by contrast, men feel uncomfortable especially when perceiving them as compliments (Holmes, 1993). Gender differences have also been observed for accuracy of facial expression recognition and results mostly indicate better performances of women regardless of the displayed emotion (Thayer and Johnson, 2000; Hall and Matsumoto, 2004; Guillem and Mograss, 2005) and allegedly documenting the superiority of women in social communicative skills. However, several studies did not replicate these differences (for review see Kret and De Gelder, 2012) suggesting that the discrepancy between women and men might be task-related. In fact, in simple emotion recognition tasks with intense facial expressions women and men show similar performances (Lee et al., 2002; Hoheisel et al., 2005; Habel et al., 2007). In addition, women tend to exhibit better performances in recognizing self-conscious emotions (e.g., pride, Tracy and Robins, 2008) whereas men seem to be faster in recognizing anger (Biele and Grabowska, 2006).

Gender differences have not only been examined on subjective but also on physiological measures. Facial electromyography (EMG) research has shown rapid and spontaneous mirroring of emotional expressions in static facial displays (Buck, 1984; Dimberg, 1997) and may therefore contribute to answering questions regarding gender differences. Specifically, positive facial expressions such as happiness evoke increased zygomaticus major muscle activity (lifting the lips to smile) in contrast to negative facial expressions such as anger which elicit increased corrugator supercilii muscle activity (responsible for frowning; Dimberg, 1990). Research examining basic emotions by using short video clips, revealed that the corrugator muscle showed increased activity to expressions of anger, sadness, and disgust, and pronounced relaxation toward happy expressions (Hess and Blairy, 2001). Several studies investigating gender differences utilized static emotional faces and found that women generally exhibited greater facial EMG responses which were most pronounced to positive facial expressions (Dimberg and Lundquist, 1990). In contrast, research using general emotional images from the International Affective Picture System (IAPS; Lang et al., 1997) did not show differences in facial EMG activity between genders (Bianchin and Angrilli, 2012). However, it is important to note that research has also put forward a dynamic facial expression approach to better represent social encounters and observed greater emotion consistent EMG activity to dynamic as compared to static expressions (e.g., Weyers et al., 2006). Importantly, dynamic facial anger expressions of avatars elicited increased corrugator muscle activity in male perceivers whereas dynamic facial happy expressions elicited higher zygomaticus muscle activity in female perceivers (Soussignan et al., 2013).

Two relevant aspects have largely been neglected in the research reviewed above. First, the perceiver and the expresser perspective have rarely been considered jointly (Biaggio, 1989; Lee et al., 2002; Hall and Matsumoto, 2004; Guillem and Mograss, 2005; Bianchin and Angrilli, 2012; Carré et al., 2013). Obtaining a complete picture of social interactions requires fully crossing perceiver and expresser gender in a 2 (perceiver gender) × 2 (expresser gender) design. Second, static images of emotional facial expressions lack the dynamic complexity of naturalistic social-emotional interactions and therefore have limited external validity. Interestingly, Kret et al. (2011) showed increased brain activation in a widespread network including the fusiform gyrus, superior temporal sulcus, and the extrastriate body area in men compared to women by using different stimulus material such as postures vs. faces. Male observers showed these increased activation patterns particularly when exposed to threatening vs. neutral male body postures but not to facial expressions. This study evidenced the importance of considering the interaction of gender and specific stimulus types in emotion perception research. In line with this, some researchers have recently called for ‘more naturalistic stimuli’ and that ‘taking into account the sex of the actor could provide further insight into the issues at stake’(Kret and De Gelder, 2012, p. 1212). Addressing these aspects we have recently developed a naturalistic video set (Blechert et al., 2013) aiming at maximizing external validity within the laboratory context. This video set (termed *E.Vids*) is balanced in gender to facilitate perceiver gender × expresser gender studies. Emotional valence-specific subjective, facial, and neural (electrocortical, hemodynamic) responses have been documented for this video set (Blechert et al., 2013; Reichenberger et al., 2015; Wiggert et al., 2015).

Based on previous findings, we expected that videos with negative expressions of male actors compared to female actors will be rated as more unpleasant and arousing by both female and male perceivers (Blechert et al., 2013; Reichenberger et al., 2015; Wiggert et al., 2015). In contrast, we expected that videos with positive expressions of female actors compared to male actors will be rated as more pleasant and arousing by both female and male perceivers. To the degree that these experiential effects translate into specific facial expressions (Rinn, 1984; Cacioppo et al., 1992; Bunce et al., 1999; Neumann et al., 2005), more positive valence ratings should be reflected by increased zygomaticus muscle activity and more negative valence should be reflected by increased corrugator muscle activity. Moreover, these effects may be modulated by perceiver gender. For instance, women may respond more negatively to negative evaluations delivered by men. Likewise, positive evaluations may be perceived as more pleasant and arousing when expressed by the opposite actor gender, both contributing to a three-way Emotion condition × perceiver gender × expresser gender interaction effect. The present study allows for a reexamination of gender differences in response to neutral and negatively valenced social stimuli (mainly based on static images). Furthermore, this study extends previous research by including positively valenced and naturalistic stimuli in a fully crossed, participant gender X stimulus gender design. Finally, following a multi-method approach, both experiential as well as facial-muscular responses are collected.

## Materials and Methods

### Participants

A sample of 58 participants (32 female) with an average age of 22.9 years (*SD* = 2.5) was recruited through online advertisement and in psychology classes. Participants reported no current mental or neurological disorders, no current use of prescriptive medication except contraceptives, and no current alcohol or drug dependence. Men and women did not differ in age, years of education or body mass index (BMI), *ts(*56) < 1.13, *p*s > 0.061. Eligible participants read and signed a consent form that was approved by the ethics committee of the University of Salzburg and received monetary compensation or course credit for participation.

### Video Set

The *E.Vids* video set (Blechert et al., 2013) comprises 3000 ms duration videos of eight negative, eight neutral, and eight positive sentences delivered by 20 actors (10 female) alongside the respective facial and gestural expressions in a naturalistic untrained manner. Negative sentences were chosen to express social criticism/disapproval (e.g., “I hate you”, “You are embarrassing!”), whereas positive sentences were chosen to express compliments and approval (e.g., “I’m proud of you”, “You’ve got it!”) and neutral sentences express neutral conditions (e.g., “It’s 4 o’clock.”, “The train goes fast.”). Expressers were instructed to act spontaneously and naturalistically and to speak directly to the camera to facilitate the perception of a real interaction in observers. Each video started with a neutral facial expression, which transitioned into the sentence with an associated facial expression after an average of 602.50 ms (*SD* = 220.32 ms). The present study utilized all sentences of 18 of the 20 expressers of *E.Vids.*

### Stimulus-Condition Assignment

In research with static faces multiple expresser identities are used for displaying different basic emotions. The emotional condition matches the expresser identity with relevance for *emotion recognition* (e.g., Phillips et al., 1998; Goeleven et al., 2008). It may reasonably be argued that assessing *emotion reactivity* should incorporate unequivocal condition and expresser assignment. Thus, in the present task, a given expresser was always (and repeatedly, but different sentences) presented within one emotional condition (negative or neutral or positive) for a given participant but expressers ‘cycle’ through conditions across participants (Pejic et al., 2013; Hermann et al., 2014) to avoid confounding expresser identity with emotional condition. Another unique feature of the present stimulus set is that the sentences spoken by a given expresser within one condition vary for a given perceiver (five out of eight sentences). This allows us to create a more varied and supposedly more capturing/naturalistic experience of the stimuli. The present passive viewing task included 90 different expresser/sentence combinations in 30 neutral, 30 negative, and 30 positive videos. In each of these three conditions, each perceiver watched six different expressers (three male) delivering five different sentences (to validate the whole stimulus set a different set of five sentences were drawn from the eight sentences available for each condition so that, across perceivers each sentence was presented with equal frequency).

### Procedure

The laboratory assessment started with sensor application for peripheral physiological measurements followed by a 4-min quiet sitting baseline and a 3-min heartbeat perception phase (results not reported here). Before the start of the task, perceivers (participants) were asked to imagine a real interaction with the displayed expressers. This was done to facilitate emotional engagement with the stimuli (Blechert et al., 2012, 2015). The 90 three-second videos were presented on a 23-inch LCD monitor with a resolution of 1920 × 1080 pixel and 120 Hz refresh rate, using E-Prime 2.0 Professional (Psychology Software Tools, Inc., Sharpsburg, PA, USA). The intertrial interval varied randomly between 5600 and 6400 ms. Video volume (delivered via external active speakers (X-140 2.0 PC-speaker system 5 W RMS, Logitech, Apples, Switzerland) was constant across perceiver. After completion of the task and sensor removal, perceiver completed several questionnaires and were then debriefed and compensated (10€) for participation.

### Self – Report Measures

Valence and arousal self-reports were assessed via a horizontal on-screen visual analog scale (“How would you feel meeting this person?”). Immediately following each video, perceivers were asked to rate their emotional response to the stimulus by indicating (un)pleasantness (0 = pleasant to 100 = unpleasant) and arousal (0 = calm to 100 = aroused/excited).

### Psychophysiological Measures: Recording, Oﬄine Analysis, and Response Scoring

Psychophysiological data were recorded with a REFA 8-72 digital amplifier system (TMSi) with 24 bits resolution at 400 Hz, streamed to disk and displayed on a PC monitor for online monitoring of data quality. Facial EMG electrodes for the bipolar recording of the corrugator supercilii and zygomaticus major activities were placed according to international guidelines (Fridlund and Cacioppo, 1986) on the left side of the face. *Oﬄine data inspection* and manual artifact rejection for EMG was done in ANSLAB 2.6, a customized software suite for psychophysiological recordings (Wilhelm et al., 1999; Wilhelm and Peyk, 2005). EMG preprocessing comprised a 28 Hz high-pass filter, a 50 Hz notch filter, rectification, low pass filtering (15.92 Hz), and a 50 ms moving average filter. Responses were defined as averages across the 3000 ms of the video plus one second after video-end (interval before ratings, since preliminary analyses revealed continued responding after video offset) referenced to a 2000 ms baseline immediately before start of the video. Separate averages were created for all positive, negative, and neutral videos as well as for expresser gender.

### Data Reduction and Statistical Analysis

Subjective ratings of valence and arousal were analyzed in two separate 2 (Expresser gender: male vs. female) × 3 (Condition: positive, neutral, negative) × 2 (Perceiver gender: male vs. female) repeated measures analyses of variance (ANOVAs) with perceiver gender as a between subject factor. The EMG measures of the corrugator and the zygomaticus muscle activity were submitted to two separate repeated measures ANOVAs as described for subjective ratings. The alpha level for all analyses was set to 0.05 and significant main or interaction effects were followed up using pairwise comparisons for repeated measure designs applying the Sidák correction (Mean differences = *MeanDiff*, significance levels, and 95% confidence intervals (CI) are displayed). Effect sizes are reported as partial eta squared $\eta_{p}^{2}$. When sphericity assumption was violated in ANOVAs, the Greenhouse-Geisser correction for repeated measures was applied with nominal degrees of freedom and epsilon ε being reported. All statistical analyses were performed using PASW Statistics 21 (SPSS Inc., Chicago, IL, USA).

## Results

### Self-Report Measures

#### Valence

**FIGURE 1:**
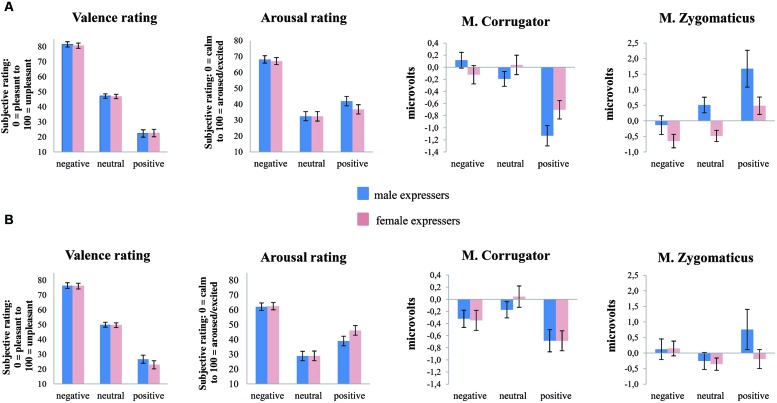
**(A)** Shows response patterns of female participants for valence and arousal ratings as well as M. corrugator and M. zygomaticus activity as a facial expressive response to emotion-evocative video-clips (negative, neutral, positive). **(B)** Shows response patterns of male participants for valence and arousal ratings as well as M. corrugator and M. zygomaticus activity. Line bars indicate standard error.

The 2 (Expresser gender: male vs. female) × 3 (Emotion condition: negative, neutral, positive) × 2 (Perceiver gender: male vs. female) repeated measures ANOVA of valence revealed a main effect of Expresser gender, *F*(1,56) = 4.16, *p* = 0.046, $\eta_{p}^{2}$ = 0.07, with male expressers being perceived as more unpleasant than female expressers (*MeanDiff* = 0.92, *p* = 0.046, 95% CI_male expresser-female expresser_ [0.017, 1.83]). As expected from previous research with this stimulus set, there was a main effect of Emotion condition, *F*(2,112) = 351.00, *p* < 0.001, $\eta_{p}^{2}$ = 0.86, ε = 0.69. Negative videos were rated as more unpleasant than neutral videos, which in turn were rated as more unpleasant than positive videos (*MeanDiffs* > 24.80, *p*s < 0.001, 95% CI_neg-neu_ [25.80, 34.69], 95% CI_neu-pos_ [20.89, 28.72]; **Figures 1A,B**). However, no main effect of Perceiver gender, *F*(1,56) = 0.003, *p* > 0.05 and no interactions of Expresser gender x Emotion condition or Perceiver gender × Emotion condition, *Fs* < 2.16, *p*s > 0.121, emerged.

#### Arousal

The ANOVA of arousal ratings revealed a significant Emotion condition effect, *F*(2,108) = 100.52, *p* < 0.001, $\eta_{p}^{2}$ = 0.65 showing that negative videos were rated as more arousing than positive and neutral videos (*MeanDiffs* > 23.79, *ps* < 0.001, 95% CI_neg-neu_ [27.99, 41.20], 95% CI_neg-pos_ [18.24, 29.35]). In addition, negative and positive videos were rated as more arousing than neutral videos (*MeanDiffs* > -34.60, *p*s < 0.001, 95% CI_neu-neg_ [-41.20, -27.99], 95% CI_neu-pos_ [-17.05, -4.56]) indicating that emotional videos elicit more arousal than neutral videos. Moreover, a significant two-way interaction by Expresser gender × Perceiver gender *F*(1,54) = 12.00, *p* = 0.001, $\eta_{p}^{2}$ = 0.18 as well as a significant three-way interaction of Expresser gender × Emotion condition × Perceiver gender, *F*(2,108) = 9.63, *p* < 0.001, $\eta_{p}^{2}$ = 0.15, ε = 0.88, emerged. In line with our hypotheses, follow-up analyses showed that female perceivers rated positive videos of male expressers as more arousing than those of female expressers (*MeanDiff* = 5.28, *p* = 0.014, 95% CI_male expresser-female expresser_ [1.12, 9.43]; **Figure 1A**) with a reverse pattern in male perceivers: they rated positive videos of female expressers as more arousing than those of male expressers (*MeanDiff* = 7.14, *p* = 0.002, 95% CI_male expresser-female expresser_ [2.68, 11.60]; **Figure 1B**).

### Facial EMG

#### Corrugator Supercilii Muscle

The 2 (Expresser gender: male vs. female) × 3 (Emotion condition: negative, neutral, positive) × 2 (Perceiver gender: male vs. female) repeated measures ANOVA revealed a significant Emotion condition effect, *F*(2,110) = 24.24, *p* < 0.001, $\eta_{p}^{2}$ = 0.31, with positive videos eliciting consistent corrugator muscle relaxation in both perceiver genders (*MeanDiffs* > -0.73, *p*s < 0.001, 95% CI_pos-neg_ [-0.94, -0.32], 95% CI_pos-neu_ [-1.02, -0.44]) relative to the other two conditions which in turn were not different from each other (*MeanDiff* = -0.10, *p* = 0.676). Moreover, significant Emotion condition × Perceiver gender, *F*(2,110) = 3.13, *p* = 0.048, $\eta_{p}^{2}$ = 0.05, and Emotion condition × Expresser gender interactions, *F*(2,110) = 6.78, *p* = 0.002, $\eta_{p}^{2}$ = 0.11, occurred. Both two-way interactions were qualified by a three-way interaction, *F*(2,110) = 4.37, *p* = 0.015, $\eta_{p}^{2}$ = 0.07 (**Figures 1A,B**). The three-way interaction was due to stronger condition effects in female perceivers, particularly when confronted with male expressers: Only in this combination (female perceivers/male expressers) all three conditions reliably differed (*MeanDiffs* > -1.25, *p*s < 0.001, 95% CI_pos-neg_ [-1.71, -0.79], 95% CI_pos-neu_ [-1.34, -0.54]) with an increase from positive to neutral to negative evaluations.

In contrast, male perceivers did not show different corrugator muscle responses for male vs. female expressers (*MeanDiff* = -0.06, *p* = 0.479) but different condition responses were also found in male perceivers, *F*(2,50) = 10.16, *p* < 0.001, $\eta_{p}^{2}$ = 0.29, with unexpected larger corrugator relaxation to positive compared to neutral videos (*MeanDiff*= 0.63, *p* = 0.002, 95% CI_neu-pos_ [0.19, 1.05]). In sum, corrugator activity suggested that male expressers elicit linear and strong emotion effects in female perceivers, with an overall special role for positive sentences (**Figure 1A**).

#### Zygomaticus Major Muscle

This pattern was partially mirrored by zygomaticus activity, the 2 (Expresser gender: male vs. female) × 3 (Emotion condition: negative, neutral, positive) × 2 (Perceiver gender: male vs. female) repeated measures ANOVA revealed a significant Emotion condition effect, *F*(2,110) = 6.70, *p* = 0.004, $\eta_{p}^{2}$ = 0.11, ε = 0.81, indicating that positive videos elicited smiling in both perceiver genders (*MeanDiffs* > 0.81, *ps* < 0.026, 95% CI_pos-neg_ [0.08, 1.55], 95% CI_pos-neu_ [0.15, 1.51]) relative to the other two conditions which in turn were not different from each other (*MeanDiff* = 0.01, *p* = 1.00) The significant Emotion condition × Perceiver gender interaction, *F*(2,110) = 3.38, *p* = 0.048, $\eta_{p}^{2}$ = 0.06, ε = 0.81 revealed that female perceivers showed more reliable and condition consistent zygomaticus responses (paralleling corrugator muscle findings) than male perceiver. Pairwise comparisons revealed that female perceivers’ zygomaticus muscle activity showed increasing responses from negative to positive and from neutral to positive conditions, irrespective of expresser gender (*MeanDiffs* > 1.07, *ps* < 0.018, 95% CI_pos-neg_ [0.48, 2.47], 95% CI_pos-neu_ [0.15, 1.99]; **Figure 1A**). In male perceivers Emotion condition effects did not reach significance (*MeanDiffs* < 0.59, *p* > 0.311) regardless of expresser gender. The Emotion condition × Expresser gender interaction, *F*(2,110) = 5.47, *p* = 0.009, $\eta_{p}^{2}$ = 0.09, ε = 0.84 revealed that positive videos of male expressers triggered enhanced smiling responses in both perceiver genders in the positive video condition relative to neutral or negative videos (*MeanDiffs* > 1.09, *ps* < 0.020, 95% CI_pos-neg_ [0.29, 2.16], 95% CI_pos-neu_ [0.14, 2.04]; **Figures 1A,B**) which was underlined by the main effect of Expresser gender, *F*(1,55) = 11.84, *p* = 0.001, $\eta_{p}^{2}$ = 0.18. However, the three-way interaction was not significant, *F*(2,110) = 0.83, *p* = 0.440.

None of the dependent variables were significantly correlated (all *ps* > 0.05).

## Discussion

Gender differences are biologically and culturally influenced (Rudman and Glick, 2010) and multiple different approaches have been put forward for their explanation. However, a large number of inconsistent findings challenge the test of their respective validity. The current study aimed to contribute to further clarify this issue. We addressed several limitations of previous research by studying social interactions considering both the expresser and the perceiver gender using a well validated, naturalistic emotion-evocative, social-evaluative video set.

### Self-Report Data

In line with our prediction, we found an opposite sex preference for positive sentences (compliments/approval) on arousal ratings supporting an unequivocal interpretation that both genders are more open to such evaluation when expressed by the opposite sex, even if these are not explicitly sexual in nature. This result is in line with previous research in the context of gender differences and positive emotions (e.g., Hofmann et al., 2006). Thus, an arousal effect by the opposite gender is generally consistent with the biological evolutionary approach emphasizing that mating strategies supporting reproduction influence positively valenced communication between the sexes (cf., Darwin, 1871). Possessing positive traits, expressing them, and perceiving them in potential opposite-sex mates have evolutionary significance since they predict successful partnership and can be passed on to the offspring. Positive statements of the opposite gender elicit excitement and associated physical arousal may support effort of approach. Interestingly, valence ratings did not show this distinct pattern of gender differences, possibly because ratings of pleasantness of expresser videos were more influenced by idiosyncratic preferences. However, valence ratings underlined the expected emotional condition effects in both perceiver genders indicating more subjective pleasantness toward positive evaluations and more unpleasantness toward negative evaluations.

### EMG Responses in the Context of One’s Own Gender Evaluations

Corrugator and zygomaticus muscles showed distinct activity patterns of perceiver genders in relation to expresser genders. In the context of one’s own gender, female perceivers exhibited an increasing corrugator response from positive to negative evaluations of female expressers. This was partially mirrored by the zygomaticus activity decreasing from positive to negative female evaluations. Similarly, male perceivers exhibited an analog pattern from positive to negative evaluations of male expressers. Both perceiver genders showed the expected valence consistent zygomaticus response to positive evaluations (smiling). This suggests that positive evaluations conveying acceptance and appreciation may elicit positive emotions such as happiness and pride which in turn may elevate self-esteem (Fleming and Courtney, 1984). However, both perceiver genders did not display the expected “frowning” response of the corrugator muscle to negative evaluations of their own gender. According to research of *emotional mimicry* which is conceptualized as a tendency to imitate the emotional expression of interaction partners particularly when people are motivated to bond with each other (Hess and Fischer, 2013), positive emotion displays are assumed to be mimicked whereas facial expressions perceived as offensive, are not mimicked (Fischer et al., 2012). In the present study, a happy face may have been mimicked by the perceiver because it was accompanied by positive evaluations which underline an affiliative intention. In contrast, negative evaluations of one’s own gender may have been considered as particularly hostile leading to an inhibition of facial responding.

### EMG Responses in the Context of Opposite Gender Evaluations

Interestingly, in the context of opposite gender evaluations, female perceivers were most responsive to male expressers, with corrugator activity increase from positive to neutral and from neutral to negative. This was further supported by the zygomaticus activity indicating an activity increase from negative to neutral and from neutral to positive evaluations. This is in line with prior research suggesting that facial expressions have been associated with higher emotional responses to happiness and anger in female than male perceivers (Biele and Grabowska, 2006). The evolutionary approach (Darwin, 1871) may point toward a particular female sensitivity to affective states of potential male partners and future caregivers. Women may respond more accurately and faster to anger expressions of men because the often physically stronger men may represent greater threats than women (for an overview see, Rudman and Glick, 2010). Additionally, female perceivers have been reported to exhibit an enhanced corrugator muscle activity compared to men when exposed to anger-eliciting stimuli (Schwartz et al., 1980; Kring and Gordon, 1998; Bradley et al., 2001). In contrast, other studies have shown that men respond faster and more precisely to anger eliciting stimuli specifically when those are posed by other males (Goos and Silverman, 2002; Seidel et al., 2010). This is incongruent to the present finding of male perceivers who only responded differentially to positive evaluations regardless of the expresser gender. This particular finding may suggest that compliments expressed by men are scarce in Western societies (Holmes, 1993) and therefore it may have demonstrated a more pleasant and surprising event leading to increased smiling by both perceiver genders toward positive social evaluations of male expressers.

According to the more distinctively emotional facial muscular responses in female than male participants, women were overall more emotionally responsive than men. This is in line with our expectation and the majority of studies investigating gender differences in emotionality using EMG and facial expressions (e.g., Greenwald et al., 1989; Thunberg and Dimberg, 2000; Bradley et al., 2001). Furthermore, according to the biological approach, those gender differences of responsiveness may also be due to differences in *emotional contagion* which is defined as “catching another person’s emotion” (Hatfield et al., 1993), automatically mimic this emotion, and in turn through interoceptive feedback mechanisms also feeling this emotion (Flack, 2006). Positive associations between facial imitative responses and empathy have been revealed in previous research (Sonnby-Borgstrom, 2002; Sonnby-Borgstrom et al., 2003) where women tend to exhibit higher empathy scores than men (Baron-Cohen and Wheelwright, 2004; Rueckert and Naybar, 2008; Derntl et al., 2010).

Unexpectedly, male expressers of positive social evaluations elicited higher responses (on corrugator and zygomaticus activity), specifically when perceived by women (corrugator activity). This result is contrary to our expectation and previous findings showing faster responses in women to angry male faces (Rotteveel and Phaf, 2004) and stronger responses and activation patterns in specific brain areas (e.g., ACC, visual cortex) in men to threatening male stimuli (Mazurski et al., 1996; Fischer et al., 2004b). However, Seidel et al. (2010) showed that happy male faces were rated more positively than happy female faces, in contrast to angry and disgusted male faces that were rated more negatively than female faces. Our current subjective ratings do not match those previous findings but facial muscle activity partially matches this set of prior subjective results. Although such discrepancies are commonplace and not always well understood, they point to the fact that much of our non-verbal communication is not well represented in our conscious experiential systems. This indicates that some populations might show dysfunctional facial-communicative behavior without explicitly being able to report or become aware of this discrepancy, leading to ambivalent or disturbing expressions or perceptions. Concordance between self-report and psychophysiological measures is often low which highlights the importance of assessing variables from both domains in emotion research (Evers et al., 2014).

It has been suggested that women are generally more emotionally expressive than men (Kring and Gordon, 1998) but as reviewed above, angry male faces tend to elicit stronger responses in both genders. According to our results, male expressers eliciting stronger responses is not limited to negative social evaluations but encompasses positive social encounters as well. Stimulus differences may explain the extended finding in the positive emotion condition. Prior research predominantly utilized basic facial emotions, thus disregarding social environments/contexts and higher-order emotions such as pride, appreciation or embarrassment. However, research has shown that gender differences in expressive behavior are context-dependent, socialized due to display rules, and emotion-dependent (for review, Kret and De Gelder, 2012). The majority of experienced emotions in our daily lives occurring in social interactions appear to be dynamic and multifaceted in nature rather than static and similar. Hence, our study is emphasizing naturalistic, dynamic stimuli with multimodal expressions (speech, gesture, and movements).

## Limitations

Our study has several limitations. We did not assess sexual orientation of participants. Furthermore, assessing contraceptive use and cycle phase in women, which have been associated with emotion recognition (Derntl et al., 2008, 2013) may further clarify variances in emotion reactivity in women. Additionally, the measurement of more facial EMG channels could give further insight to the involvement of specific emotions (for review see, Hess and Fischer, 2013). Future research may utilize this stimulus set for facial action coding (Ekman and Friesen, 1978) to more precisely map emotion expressions relating to social interactions. In this context we cannot rule out differential cognitive emotion regulation strategies in men and women. It is generally difficult in this type of research to disentangle emotion reactivity due to emotional contagion from emotional mimicry. Furthermore, the sample here was chosen to match age of the actors. Language of the stimuli was age – appropriate for university students between 20 and 30 years. Thus, the present results are probably more applicable to this age group and to peer – interaction. Other age groups or between generation interaction might well show different response patterns. This limits the generalization of the results and points to new avenues of research.

## Conclusion

In summary, the current study contributes to further clarification of gender differences in emotional social interactions utilizing a more ecologically valid and naturalistic paradigm. Specifically, in the positive social evaluation condition, valence congruent facial muscular responses of both perceiver genders have been displayed. Furthermore, this study takes the first step in revealing pronounced positive expressive communication patterns in men (male expressers) during social interactions. Therefore, gender differences in positive social encounters associated with both perspectives (perceiver and expresser) deserve more attention in future research.

## Conflict of Interest Statement

The authors declare that the research was conducted in the absence of any commercial or financial relationships that could be construed as a potential conflict of interest.
