# Supplementary material for: Gender differences in experiential and facial reactivity to approval and disapproval during emotional social interactions
Source: Front Psychol. 2015 Sep 22;6:1372. doi: 10.3389/fpsyg.2015.01372 (PMC4585028; doi:10.3389/fpsyg.2015.01372)
Supplement: Supplementary file 1 [file Data_Sheet_1.DOCX]

Supplementary Material

Gender differences in experiential and facial reactivity to approval and disapproval during emotional social interactions

Nicole Wiggert*, Frank H. Wilhelm, Birgit Derntl, and Jens Blechert

*** Correspondence:** Nicole Wiggert: nicole.wiggert@sbg.ac.at

**1 Facial EMG: Corrugator Supercilii Muscle**

Additional second by second analyses were conducted for the corrugator muscle: We used difference scores for the factor Emotion condition by subtracting neutral from negative responses (as well as neutral from positive responses) to analyze the 2 (Expresser gender: male vs. female) x 2 (Emotion condition: negative – neutral vs. positive - neutral) x 4 (Time: second by second) x 2 (Perceiver gender: male vs. female) repeated measures ANOVAs. Results revealed remaining main effects (as reported in the MS) of the Emotion condition, *F*(1, 55) = 25.22, *p* <.001, η^2^_p_ *=*.31 as well as Expresser gender, *F*(1, 55) = 5.23, *p* =.026, η^2^_p_ *=*.09 and an additional main effect of Time, *F*(3, 165) = 6.81, *p* <.001, η^2^_p_ *=*.11, ε = .57. Additionally, Emotion condition x Perceiver gender, *F*(1, 55) = 4.12, *p* =.047, η^2^_p_ *=*.07 and Emotion condition x Expresser gender, *F*(1, 55) = 7.01, *p* =.011, η^2^_p_ *=*.11 interactions occurred. Those were again qualified by the three way interaction, *F*(1, 55) = 8.24, *p* =.006, η^2^_p_ *=*.13. Moreover, including the factor Time, a significant 4-way interaction was found, *F*(3, 165) = 4.75, *p* =.003, η^2^_p_ *=*.08. The 3-way interaction of Expresser gender x Condition x Time showed also a significant effect, *F*(3, 165) = 2.69, *p* =.048, η^2^_p_ *=*.05. Post-hoc analyses for each time window (i.e., TW (1, 2, 3, and 4): seconds (1-4) showed that during TW 1 only female perceivers exhibited larger corrugator relaxation towards positive videos of male expressers (*MeanDiff* = .27, *p* =.001, 95% CI_Diff(neg-neu)-Diff(pos-neu)_ [.12, .42]) than to female expressers (*MeanDiff* = .14, *p* =.097). Male perceivers did not show this differentiation in any of the 2 conditions (*MeanDiffs* < .08, *ps* >.321). TW 2 showed that female perceivers showed larger corrugator relaxation to positive than negative videos regardless of expresser gender (*MeanDiffs* > .61, *ps* <.001, male expressers: 95% CI_Diff(neg-neu)-Diff(pos-neu)_ [1.00, 1.76], female expressers: 95% CI_Diff(neg-neu)-Diff(pos-neu)_ [.26, .98]) whereas male perceivers did only show this differentiation to positive videos of male expressers but not female expressers (*MeanDiffs* =.49, *p* =.021, 95% CI_Diff(neg-neu)-Diff(pos-neu)_ [.08, .91]). TW 3 showed similar patterns for female perceivers as in TW 2, (*MeanDiffs* > .81, *ps* <.006, male expressers: 95% CI_Diff(neg-neu)-Diff(pos-neu)_ [1.26, 2.22], female expressers: 95% CI_Diff(neg-neu)-Diff(pos-neu)_ [.24, 1.39]). However, male perceivers did not show this differentiation, (*MeanDiffs* < .58, *ps* >.066). TW 4 showed the same corrugator muscle activity differentiation as in TW 1 with only female perceivers exhibited larger corrugator relaxation towards positive videos of male expressers (*MeanDiff* = 1.36, *p* <.001, 95% CI_Diff(neg-neu)-Diff(pos-neu)_ [.81, 1.90]) but to female expressers (*MeanDiff* = .44, *p* =.160). Male perceivers did not show this differentiation in any of the 2 conditions (*MeanDiffs* < .08, *ps* >.321).

**2 Facial EMG: Zygomaticus Major Muscle**

Additional second by second analyses were conducted for the zygomaticus muscle: We used difference scores for the factor Emotion condition by subtracting neutral from negative responses (as well as neutral from positive responses) to analyze the same ANOVA design as for the corrugator muscle. Results revealed a remaining main effect (as reported in the MS) of the Emotion condition, *F*(1, 55) = 8.73, *p* =.005, η^2^_p_ *=*.14 and an additional main effect of Time, *F*(3, 165) = 11.85, *p* <.001, η^2^_p_ *=*.18, ε = .41. Both interactions of Emotion condition x Perceiver gender, *F*(1, 55) = 5.17, *p* =.027, η^2^_p_ *=*.09 and Emotion condition x Expresser gender, *F*(1, 55) = 12.97, *p* =.001, η^2^_p_ *=*.19 remained significant. Additionally, a significant 3-way interaction of Expresser gender x Emotion condition x Time, *F*(3, 165) = 6.39, *p* <.001, η^2^_p_ *=*.10, ε = .66 was shown. Post-hoc analyses for each time window (i.e., TW same as for the corrugator analysis) showed that during TW 1 no differences between expresser genders in any condition appeared (*MeanDiffs* < .15, *ps* >.358). However, TW 2 showed that male and female expressers elicited more zygomaticus muscle activity in the positive compared to the negative condition (*MeanDiffs* > .53, *ps* <.002, male expressers: 95% CI_Diff(neg-neu)-Diff(pos-neu)_ [-1.45, -.34], female expressers: 95% CI_Diff(neg-neu)-Diff(pos-neu)_ [-.90,- .16]) but both expresser genders did not differ from each other in the positive condition (*MeanDiff =*.26, *p* =.160, 95% CI_male expresser – female expresser_ [-.11, .62]). TW 3 revealed a similar result pattern as TW 2 with both expresser genders eliciting more smiling in the positive compared to the negative condition, (*MeanDiffs* > .75, *ps* <.044, male expressers: 95% CI_Diff(neg-neu)-Diff(pos-neu)_ [-3.40, -.91], female expressers: 95% CI_Diff(neg-neu)-Diff(pos-neu)_ [-1.49,- .02]) and specifically male expressers elicited more smiling than female expressers in the positive condition (*MeanDiff* = 1.04, *p* =.022, 95% CI_male expresser - female expresser_ [.15, 1.92]). In TW 4 the significant differentiation that male expressers elicited more smiling in the positive condition than female expressers was confirmed, (*MeanDiff* = 1.33, *p* =.021, 95% CI_male expresser - female expresser_ [.21, 2.46]). In addition, a second 3-way interaction of Perceiver gender x Emotion condition x Time, *F*(3, 165) = 3.11, *p* =.028, η^2^_p_ *=*.05, ε = .39 appeared. No other interactions with the factor Time reached significance, *Fs*(3, 165) < 1.14, *ps* >.336. Post-hoc analyses revealed no differences between female and male perceivers in TW 1 in any condition (*MeanDiffs* < .36, *ps* >.086). TW 2, TW 3, and TW 4 showed that female perceivers showed more smiling in the positive condition, irrespective of expresser gender (*MeanDiffs* > 1.07, *ps* <.036) whereas male perceivers did not show this differentiation (*MeanDiffs* < .81, *ps* >.120).
